# Genome-Wide Association Studies on Resistance to Pea Weevil: Identification of Novel Sources of Resistance and Associated Markers

**DOI:** 10.3390/ijms25147920

**Published:** 2024-07-19

**Authors:** Salvador Osuna-Caballero, María J. Cobos, Carmen M. Ruiz, Osman Z. Wohor, Nicolas Rispail, Diego Rubiales

**Affiliations:** Institute for Sustainable Agriculture, Spanish National Research Council (CSIC), Av. Menéndez Pidal s/n, 14004 Córdoba, Spain

**Keywords:** *Bruchus*, GWAS, *Pisum*, resistance breeding

## Abstract

Little resistance to the pea weevil insect pest (*Bruchus pisorum*) is available in pea (*Pisum sativum*) cultivars, highlighting the need to search for sources of resistance in *Pisum* germplasm and to decipher the genetic basis of resistance. To address this need, we screened the response to pea weevil in a *Pisum* germplasm collection (324 accession, previously genotyped) under field conditions over four environments. Significant variation for weevil seed infestation (SI) was identified, with resistance being frequent in *P. fulvum*, followed by *P. sativum* ssp. *elatius*, *P. abyssinicum*, and *P. sativum* ssp. *humile*. SI tended to be higher in accessions with lighter seed color. SI was also affected by environmental factors, being favored by high humidity during flowering and hampered by warm winter temperatures and high evapotranspiration during and after flowering. Merging the phenotypic and genotypic data allowed genome-wide association studies (GWAS) yielding 73 markers significantly associated with SI. Through the GWAS models, 23 candidate genes were found associated with weevil resistance, highlighting the interest of five genes located on chromosome 6. These included gene 127136761 encoding squalene epoxidase; gene 127091639 encoding a transcription factor MYB SRM1; gene 127097033 encoding a 60S ribosomal protein L14; gene 127092211, encoding a BolA-like family protein, which, interestingly, was located within QTL *BpLD.I*, earlier described as conferring resistance to weevil in pea; and gene 127096593 encoding a methyltransferase. These associated genes offer valuable potential for developing pea varieties resistant to *Bruchus* spp. and efficient utilization of genomic resources through marker-assisted selection (MAS).

## 1. Introduction

Pea (*Pisum sativum* L.) is one of the most important temperate grain legumes worldwide. Its use extends from field or dry pea to green or vegetable pea [1]. In addition, pea offers valuable environmental benefit as a legume crop, being a safe bet for sustainable agriculture. However, stored pea seeds are highly prone to severe damage by pea weevil (*Bruchus pisorum* L., Coleoptera: *Bruchidae*). Infestation starts earlier in the field, with adults feeding on pea pollen and laying seeds on young, forming pods. The emerging larvae penetrate the seeds through pods, and then, they feed on the seed endosperm during storage, devaluating seed quality and marketability [2]. Adults emerge from infested seeds in the store, compelling post-harvest fumigations to prevent movement to the fields [3]. Several approaches, including cultural, biological, physical, and chemical control measures, have been implemented with the aim of managing these pests, but none of them have been successful across time and space [4,5].

This reinforces the need to develop resistant cultivars in order to achieve a more economically and environmentally sustainable method of control. Although some efforts have been made on the development of bruchid-resistant transgenic crops, like the transfer of-amylase inhibitor from common bean to pea [6], the cultivars developed so far are yet to be commercialized worldwide because of various limitations, including raised concerns due to their potential immunogenicity [7]. Some sources of natural resistance to pea weevil are also available in germplasm [2,8,9], but these are incomplete and of complex inheritance [10,11,12].

A first genetic analysis on an F2 population from an interspecific cross between *P. sativum* × *P. fulvum* under controlled conditions identified a number of QTL associated with cotyledon resistance and seed coat or pod wall resistance [11]. A later linkage mapping on a recombinant inbred line (RIL) population derived from another interspecific cross involving *P. sativum* × *P. sativum* ssp. *syriacum* identified three QTL associated with reduced *B. pisorum* seed infestation (SI) under field conditions, with individual contribution to the total phenotypic variance ranging from 14.8 to 24% [12]. More recently, another genetic analysis using two F2 populations identified a major QTL controlling seed resistance in pea against *Callosobruchus chinensis* and *C. maculatus* [13]. These QTL techniques have been useful, although the low allelic variation contained in the bi-parental populations coupled with the low marker density in these QTL studies hindered the precision and efficiency of identifying the genetic loci associated with these traits [14]. Advanced high-throughput technologies and genomic approaches such as genome-wide association studies (GWAS) serve as a valuable complement to QTL mapping [15,16]. While the QTL confidence interval often covers large genomic regions that include numerous linked genes, GWAS can identify individual genes or nucleotides closely associated with the trait, delivering a more precise information [14,17]. The GWAS strategy aims to capture the collective effect of all loci, irrespective of their effect size, to explain the complete genetic variation of a quantitative trait in a diverse population [16,18]. Accordingly, GWAS has emerged as a powerful approach to dissect the complex genetic architecture underpinning quantitative traits such as quantitative and partial disease resistance. Coupled to a reduced-representation genome sequencing (RRGS) approach such as diversity arrays technology sequencing (DArT-Seq), which delivers a large set of molecular markers spanning the whole genome, GWAS allows scanning the whole genome for polymorphisms correlated with phenotypic variance, overcoming the constraints of bi-parental population mapping [19]. In pea, GWAS successfully aided the identification of novel variant–trait associations for breeding valuable agronomic, stress resistance, and quality attributes [18,20,21,22,23,24,25,26,27], although resistance to post-harvest pests such as pea weevil has been poorly explored so far.

The objectives of the present study were to explore the phenotypic variation for resistance to *B. pisorum* in a pea core collection across different environments, allowing the identification of new resistant sources with potential for breeding. In addition, the genetic architecture of this quantitative trait is dissected through a GWAS approach, allowing its connection to high-quality Silico-DArT polymorphic markers. Consequently, candidate genes behind weevil SI resistance in pea are proposed and their implications discussed.

## 2. Results

### 2.1. Phenotypic Response and Variance Components

A wide SI variation was observed along the four environments, with values ranging from 0% to 56% seed infestation across environments. As displayed in Table 1, the most affected environment was Puente20, with a mean and median for SI of 11.16% and 8.86%, respectively. By contrast, Agrario19 was the least affected, with a mean SI of 5.60% and a median of 4.00%. Due to excessive right skewness and a leptokurtic kurtosis in the raw data, the percentages of SI were arcsine-transformed prior to model fitting to ensure the value normalization within environments.

The linear mixed model applied allowed the obtention of the variance component within each environment (Agrario19, Agrario20, Puente19, and Puente20) and across the four environments (BLUP_MET), as shown in Table 2. Puente19 and Agrario20 displayed the greatest genetic component for SI, while Agrario19 showed the lowest heritability. In all cases, the genotypic coefficient of variation (CV_g_) was higher than the residual coefficient of variation (CV_r_), showing a CV_ratio_ > 1. The coefficient of determination of the GEI effect was high, where 33% of the SI variation was due to GEI effect. The genotype–environment correlation was also high (r_ge_ = 0.51), displaying a similar genetic response across the four environments.

**Figure 1:**
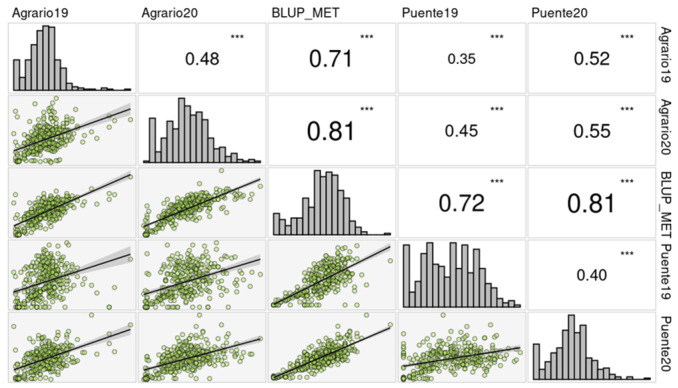
Histogram distribution and SI correlation between environments. *** indicate statistically significant correlation at 0.05, 0.01 and 0.001 level respectively.

This was also evidenced by the correlation for SI between environments (Figure 1), where the Pearson correlations of the predicted means calculated through the LMM model were all significant (*p*-value < 0.001) between environments, ranging from moderate (0.35–0.55) to high (0.71–0.81) correlations. The highest correlations were observed between BLUP_MET and the rest of the environments, ranging from 0.71 to 0.81, highlighting how the MET model is capable of merging the GEI effect into the predicted means. The highest phenotypic correlation was between Agrario20 and Puente20 (0.55), which share the same season but have different locations. By contrast, the lowest phenotypic correlation was obtained between Puente19 and Agrario19 (0.35), which also share the same field season but differ in location.

**Figure 2:**
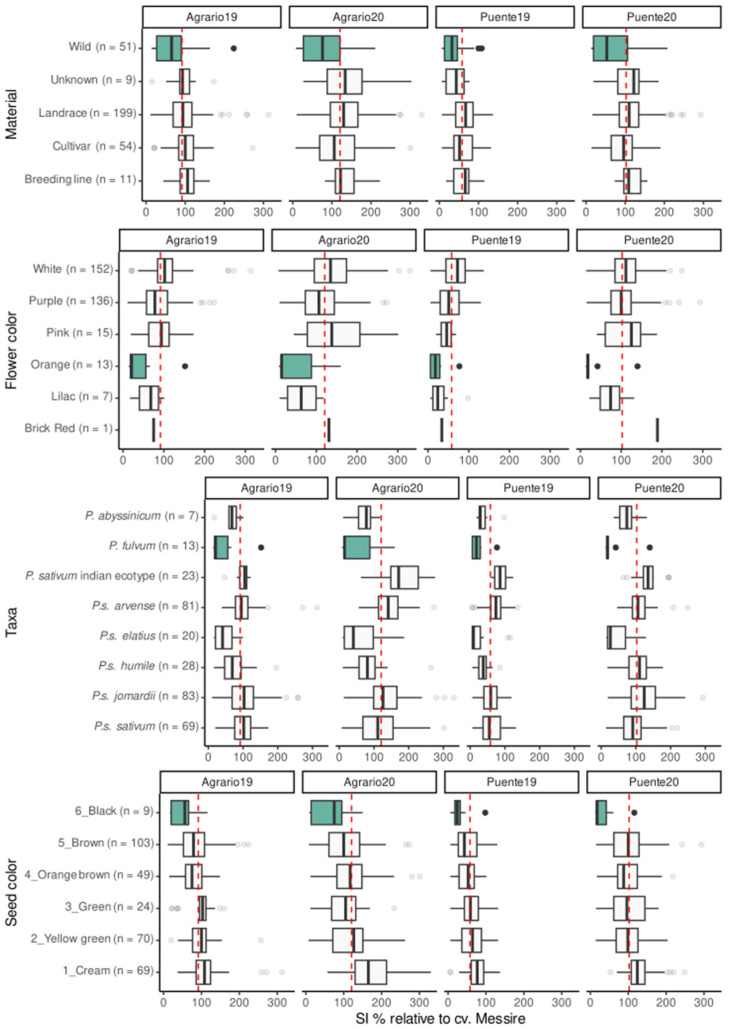
Boxplot representing the SIr (Seed Infestation relative to the control cv. Messire, established at 100%) value in each environment categorized by material type, flower color, taxa, and seed color. Red dashed lines show the average SI value of each environment. The most resistant group in each category with a *p*-value < 0.001 is highlighted in teal color.

Weevil infestation varied considerably across the diverse pea core collection, influenced by factors such as material type, flower and seed color, and taxonomic grouping, all of which affect the percentage of SI (Figure 2). Notably, *P. fulvum* accessions, characterized by orange flowers and black seeds, represent one of the most resistant groups to weevil SI within the collection across environments. Apart from the *P. fulvum* accessions, resistance was also detected in the remaining wild accessions, including the *P. sativum* subsp. *elatius* accessions and, to a lesser extent, the accessions of *P. abyssinicum* and *P. sativum* subsp. *humile*. Accordingly, black and brown seeds showed lower SI than green and yellow seeds.

### 2.2. GGE Model and Identification of New Resistant Sources

Genotype plus the genotype–environment interaction (GGE) model has been widely used for genotype evaluation in MET. The first two PC represented 81.4% of the total SI variation, where PC1 accounted for 64.5% of the variation and PC2 for 16.9%.

**Figure 3:**
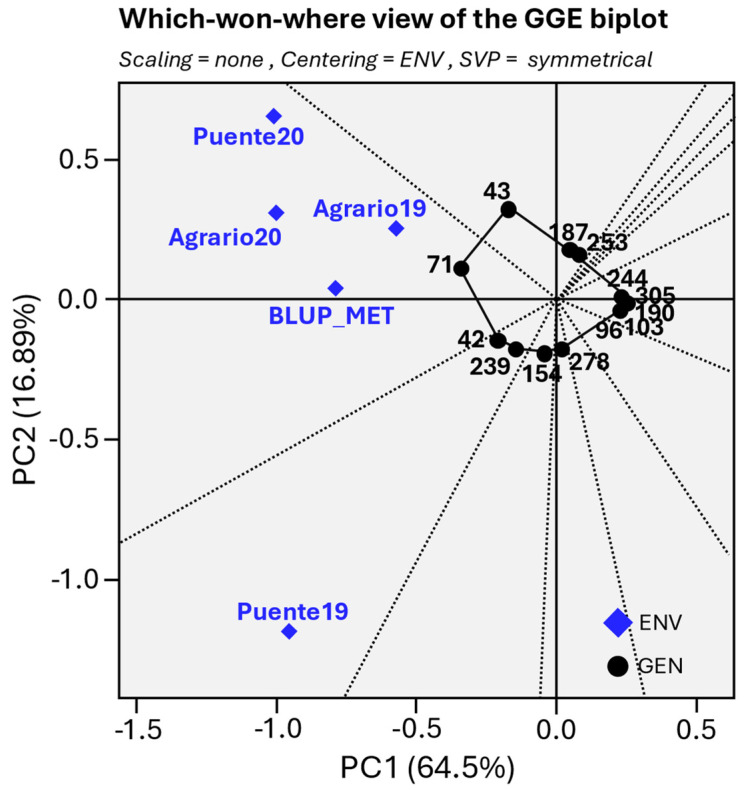
Genotype plus genotype–environment interaction biplot.

In this GGE biplot, known as which-won-where biplot (Figure 3), a polygon is drawn by joining the genotypes #43, #187, #253, #244, #305, #190, #103, #96, #278, #154, #239, #42, and #71, which are located farthest from the biplot origin, so that all other genotypes are contained in the polygon. To avoid overlapping, the genotypes contained in the polygon are not represented on the biplot (Figure 3). The vertex genotypes have the longest vectors to the origin in their respective directions, which is a measure of responsiveness to environments. Therefore, the vertex genotypes are among the most responsive genotypes. For instance, genotype #71 was the most affected by weevil in environments Puente20, Agrario20, and Agrario19, while the genotypes #42 and #239 were those with higher SI in Puente19. By contrast, genotypes #244, #305, #190, #103, and #96 are less responsive in their respective directions, showing the lowest SI and highest stability across environments (i.e., less variability between locations). The genotypes displaying the highest SI resistance and stability across environments are presented in Table 3. The full table displaying the 324 genotypes is presented in the Supplementary Material (Table S2).

**Figure 4:**
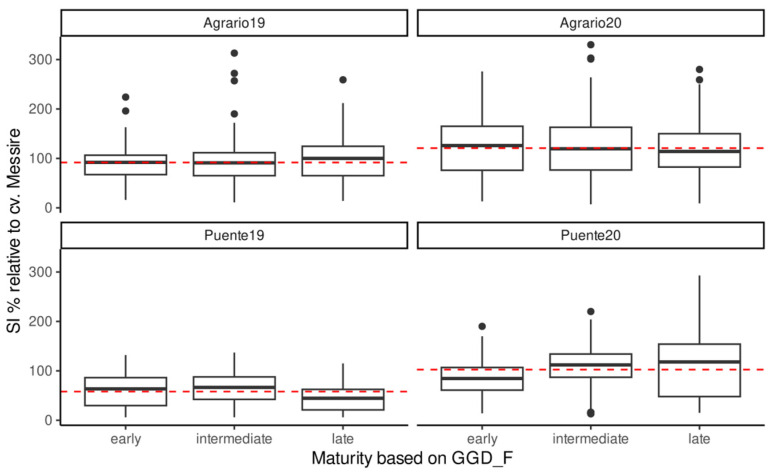
Boxplot representing the influence of maturity stage on seed infestation (SIr). Red dashed lines represent the average SIr by environment.

Regarding environments, the PC1 ranks them based on their SI, with Agrario19 as the less affected environment in contrast to Puente20 (Figure 3 and Table 1). The PC2 groups the environments into two clusters, with Puente19 being the most divergent environment. This discordance was also evident in the relationship between the precocity of pea genotypes and SIr, as shown in Figure 4. Notably, the environments that are most distant on PC2 in the GGE biplot (Figure 3) exhibit opposing trends in relation to genotype precocity (GDD_F) and SI. Specifically, in Puente19, both early and intermediate maturity genotypes suffer more significantly from weevil infestation (*p* < 0.01 and *p* < 0.001, respectively) compared to late-maturity genotypes. Conversely, in Puente20, the pattern of weevil infestation is reversed, with late-maturity genotypes experiencing higher infection levels than their early and intermediate counterparts (*p* < 0.001 and *p* < 0.01, respectively), as detailed in Figure 4.

### 2.3. Correlations between Climatic Variables and SI

**Figure 5:**
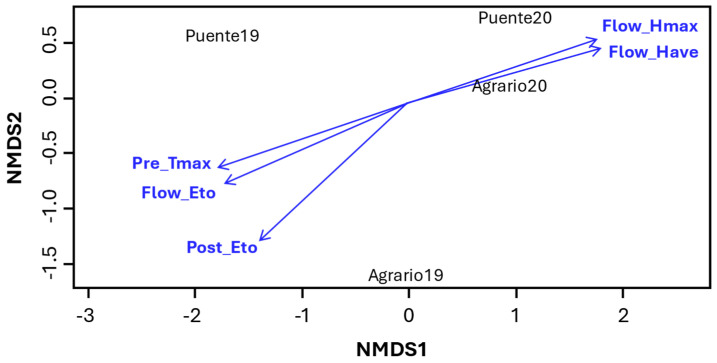
NMDS analysis. The biplot shows the climatic variables that participate in the SI variation by environment with a *p* < 0.05. These climatic variables are grouped in two clusters: (i) at the left, maximum temperature at pre-flowering (Pre_Tmax), accumulated evapotranspiration until flowering (Flow_Eto), and accumulated evapotranspiration post flowering (Post_Eto) and (ii) average humidity and maximum humidity until flowering (Flow_Have and Flow_Hmax, respectively).

The influence of environmental factors on weevil SI was calculated through a non-metric multi-dimensional scaling ordination (NMDS) analysis (Figure 5). Biplots gave a SI stress value of 0.023, indicating an excellent fit [28], which allowed the separation of environments with a clear gradation fitting level for SI. Figure 5 shows the influence of climatic variables on SI. To avoid multicollinearity, only significant climatic variables with a *p*-value < 0.05 are depicted on Figure 5. Environments at the bottom (coordinate NMDS 2) of the biplot are those with the lowest SI prevalence. The length and direction of the vectors indicate their influence on SI. The longer the vector, the greater its influence on SI, and this is negative when pointing down and positive when pointing up. Accordingly, the biplot reveals that weevil SI is enhanced by high humidity values during flowering (higher Flow_Hmax and Flow_Have) but, on the contrary, is hampered by warm winter temperatures (Pre_Tmax), excessive evapotranspiration during flowering (Flow_Eto), and post flowering (Post_Eto).

### 2.4. Positive Signals in GWAS Output

**Figure 6:**
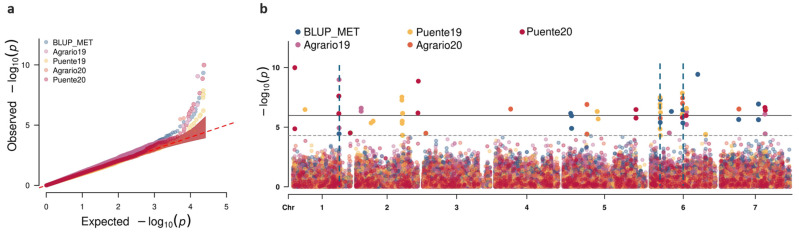
Q-Q (**a**) and Manhattan (**b**) plots of the BLINK model output. Blue dashed lines represent three genomic regions where combination of same MTAs in different environments occurred.

To identify genetic variants contributing to resistance against SI, we conducted a genome-wide association study (GWAS) employing MLM and BLINK model approaches. To account for population structure, we incorporated the Astle kinship matrix. The Bayesian information criterion (BIC) suggested that no additional covariables were required to account for the population structure of the pea collection. Inspection of the quantile–quantile (Q-Q) plots indicated appropriate model calibration, as demonstrated by the close alignment between expected and observed *p*-values, confirming the absence of genomic inflation or deflation (Figure 6a).

Using two models that applied the false-discovery rate (FDR) method (LOD = 4.65), our GWAS identified 73 markers significantly associated with SI, as detailed in Table S3. Of these, 65 markers were successfully mapped onto the *P. sativum* accession ZW6 genome and 62 onto the *P. sativum* cv. Cameor genome. Under the more stringent Bonferroni correction, with a LOD threshold of 5.72, only 43 markers were deemed significant. The spatial distribution of these markers across all seven chromosomes is depicted in a Manhattan plot for each study environment (Figure 6b). Significant marker–trait associations (MTAs) were identified on all chromosomes. Chromosomes 3 and 4 each harbored only one significant MTA, with the associated marker linked to Agrario20 SI. By contrast, the remaining chromosomes featured multiple MTAs, with three genomic regions—two on chromosome 6 and one on chromosome 1—emerging as hotspots due to their consistent associations with SI resistance across different environments. This consistency suggests these regions as promising candidates for marker-assisted selection in pea breeding programs aimed at enhancing weevil resistance.

### 2.5. Candidate Genes Involved in SI Resistance Pathways

Through the GWAS models, 23 candidate genes associated with weevil resistance (reduced SI) were identified (Table 4). The most remarkable genes are those that include more than one allelic variant that (i) explain a high portion of the phenotypic variance (i.e., high PVE), (ii) provide a strong positive signal far from the limit of detection threshold (i.e., high LOD), and (iii) are uncovered in more than one environment.

Following these criteria, five proposed genes located on chromosome 6 are of high interest. For instance, three DArT markers were linked to gene 127136761. These markers were positively associated with Puente19, Puente20, and BLUP_MET and explained from 2.7 to 13.6% of phenotypic variance (Table 4). This gene encodes a squalene epoxidase, an enzyme that is vital for the biosynthesis of cyclic triterpenoids, which are important for embryo and seed development [29,30].

At 303 kb downstream from the 127136761 gene, two positive markers were associated with all environments except Agrario19 explaining from 4.8 to 8% of the phenotypic variance (Table 4). They were linked with the gene 127091639, which encodes the transcription factor MYB SRM1, known to modulate ABA response during seed germination and seedling development under salt stress [31]. The DArT marker with the highest PVE (30.8%) was located within gene 127097033. This gene produces the 60S ribosomal protein L14 and contains another positive marker in the Agrario19 and Puente20 environments, which also explains the notable SI variation in these environments (PVE = 17.5% and PVE = 19.4%, respectively).

**Figure 7:**
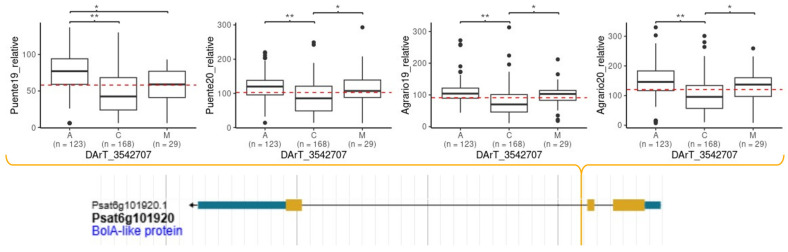
Phenotypic response of each allelic variation by environment of the marker DArT_3542707 located in the gene Psat6g101920 of the Cameor genome, corresponding with gene 127092211 (NCBI gene ID) in ZW6 genome. * and ** represent significant pair-wise differences in the level of SI between allelic variant at 0.05 and 0.01 respectively.

Interestingly, an additional marker located on chromosome 6, which explained an SI variation of 23.6% and 18.1% in BLUP_MET and Puente19, respectively co-localize within the confidence interval of QTL *BpLD.I* previously described by Aznar-Fernández et al. (2018) [9]. This DArT marker is located within an intron of gene 127092211, encoding a BolA-like family protein (Figure 7), which plays a repressive role in the tolerance against excess iron and paraquat-induced oxidative stress in plants [32]. For all the tested environments, the allelic variation with the major frequency was correlated with lower SI% in comparison to the allelic variation with the minor frequency (Figure 7). The mean SIr differences, with a *p*-value < 0.001, between the major and minor allele variant by environment were 30, 33, 33, and 53% in Puente19, Puente20, Agrario19, and Agrario20, respectively (Figure 7).

Lastly, the allelic variation of the DArT marker 3552572 also explained a high portion of the SI variance, with a PVE of 29.2, 22.9, and 15.8% in BLUP_MET, Puente19, and Puente20, respectively. This marker mapped within the candidate gene 127096593, which encodes a methyltransferase METTL6 in pea.

Several candidate genes harboring markers with individual-environment relevance were also identified. For example, DArT markers 3547098, 3542446, and 3558655 are located within gene 127119769, which encodes the heat-shock factor protein HSF8 in pea. This suggests a potential role in thermal stress responses. Furthermore, marker 4657153, which accounts for 17.3% of the PVE for SI in Agrario20, was found within gene 127105952. The function of this gene in pea remains undefined (Table 4). Additionally, two DArT markers were identified within gene 127097188, which encodes an endoglucanase 12-like protein. These markers are situated within the confidence interval of the previously identified BpSI_III QTL [9] (Table 4).

## 3. Discussion

Pea weevil is a major post-harvest pest of pea. Control of this pest is difficult, requiring periodic pesticide treatment, which is costly and poses important environmental issues. To circumvent these problems, the use of resistant accessions is of importance. Accordingly, some sources of incomplete resistance to bruchid infestations have been reported in pea [9,33], providing some levels of control, although complete control was not achieved. Therefore, there is a continuous need to identify and characterize new sources of resistance and identify molecular markers closely associated with weevil SI resistance to facilitate its introgression to elite pea cultivars. To this aim, we assessed the seed infestation by pea weevil of a pea germplasm collection under field conditions over several seasons and performed a genome-wide association study (GWAS). This allowed the identification of several novel sources of partial weevil SI resistance.

Our results show notable consistencies with previous research on the same plant material. Specifically, of the 52 wild pea accessions evaluated by Aznar-Fernández et al. (2018) [9], 42 were included in the core collection assessed in this study. Among these, the five most resistant accessions are identical in both studies, namely genotypes #267 (SIr = 48%), #306 (SIr = 54%), #313 (SIr = 63%), #251 (SIr = 69%), and #315 (SIr = 77%). These accessions belong to *P. abyssinicum*, *P. sativum* subsp. *jomardii* and *P. sativum* subsp. *elatius*, respectively (Table S2). This reinforces the findings of previous studies and highlights the value of wild crop relatives as reservoirs for disease and pest resistance traits [9,34,35]. Additionally, this study identified accessions exhibiting higher levels of resistance (i.e., lower SI and SIr) in comparison to [9], particularly among wild species. This increased resistance was predominantly found in *P. fulvum* (accessions #311, #309, #97, #305, and #308) and *P. sativum* subsp. *elatius* (accessions #249, #103, #248, #269, #246, #244, #250, and #190) and to a lesser extent in *P. sativum* subsp. *humile* (accession #110). In addition, a promising level of partial resistance was also observed in the *P. sativum* subsp. *sativum* accession #197, indicating that some domesticated pea cultivars also harbor weevil-resistance traits. These novel sources of resistance add to the limited sources of resistance available so far, facilitating resistance breeding programs (Table 4).

The resistance mechanisms harbored by these novel resistance sources are currently unknown. It is likely the result of a combination of escape, antixenosis (non-preference), and antibiosis mechanisms, resulting in reduced seed infestation and retarded larval development [9,33]. Escape by sowing date and plant phenology can affect bruchid infestation by the asynchrony between the appearance of the insect and the appropriate stage of the plant to be infested. The weevil SI escape mechanism is therefore strongly influenced by climatic condition, as we observed (Table 2 and Figure 5). This agrees with earlier reports that showed the impact of weather conditions on the survival and spread of *B. pisorum*, with adverse temperatures and high radiation reducing infection, as these might disturb oviposition and reduce egg viability [36]. Accordingly, higher *B. pisorum* or *B. rufimanus* infestation has been reported on early flowering pea and faba bean accessions, respectively [37,38,39]. While we observed such correlation in some of the environments (i.e., Puente19), it was not detected in Puente20, where the tendency was the opposite, with the late-maturity accessions being more infested (Table 2). This points to the establishment of a more favorable condition for infestation later in the season in this environment, as shown by the NMDS (Figure 5) confirming climatic condition as a main driver of weevil SI.

Antixenoxis resistance mechanisms are mediated by secreted secondary metabolites. For instance, some pea volatiles released at different phenological stages have been reported to affect the behavior of the pea weevil [36,40]. Similarly, volatiles emitted by flowers of common bean accessions resistant to Mexican weevil (*Zabrotes subfasciatus*) have been shown to have a repellent effect, resulting in adult antixenosis and reduced oviposition on seeds [41]. Pod morphological traits such as wax layers and pod thickness have also been documented to affect *B. pisorum* preference for oviposition [42,43]. Additionally, neoplasm formation has been suggested to contribute to weevil resistance [5,43]. We observed neoplasms in a few accessions in some of the environments but not all, finding neoplasm formation to be highly affected by environmental factors [44] and to have little correlation with seed infestation in the field, in agreement with Sari et al. (2020) [45]. We also observed a graduation in the level of SI according to the color of the seed coat. Accessions with lighter seed color presented higher SI (Figure 2), which agrees with reported associations of resistance with compounds in the seed coat (tannins, flavonoids, total phenolic content, and antioxidant activity) [46,47,48,49,50]. Resistance has also been associated with components of the cotyledons that can retard insect growth and development when ingested (lectins, arcelins, phyto-hemagglutinins, α-amylase inhibitors, and protease inhibitors [4,51,52]. This was not covered in our study but might be of great interest to elucidate the antibiosis component of the resistance in the identified resistant accessions. In this regard, resistance in pea was obtained by the transgenesis of the α-amylase inhibitor gene from common bean [6], as achieved in other legumes. However, these resistant materials were not adopted due to various limitations, including concerns about their potential immunogenicity [7] and the rigid legislation in some countries concerning genetically modified organisms (GMOs), coupled with low public acceptance [35].

To circumvent this limitation, resistance is being transferred to elite cultivars by conventional breeding. Marker-assisted breeding involving the identification of DNA-based markers linked to host resistance against bruchids has shown some success in the quest for the development of bruchid-resistant cultivar(s) of various legume crops. Most genetic studies on resistance to bruchids in different legumes have shown complex inheritance [35]. Various QTLs have been identified associated with resistance to cowpea weevil (*Callosobruchus maculans*) in cowpea (*Vigna unguiculata*) and chickpea (*Cicer arietinum*), to common bean weevil (*Acanthoscelides obtectus*) in common bean (*Phaseolus vulgaris*) [52,53,54,55], to *C. chinensis* and *C*. *maculatus* in mung bean (*V. radiata*) [56,57,58] and Zombi bean (*Vigna vexillata*) [59]. Several QTLs have also been described in pea for resistance to *B. pisorum*, *C. chinensis*, and *C. maculatus* [11,12,13]. Interestingly, a single major QTL, *qPsBr2*.1, was identified for *C. chinensis* and *C. maculatus* resistance. Further map-based cloning and genomic approaches on *qPsBr2.1* identified the gene Psat2g026280 (designated as PsXI), which encodes a xylanase inhibitor as the underlying gene responsible for bruchid resistance [13] in this mapping population. By contrast, Aznar-Fernández et al. (2020) [12] described three QTLs associated to reduced SI and an additional one for reduced larval development in response to *B. pisorum*. However, the large confidence interval of these QTLs spanning several Megabase pairs impeded the identification of candidate genes. Our GWAS approach detected 73 markers associated with SI variation in at least one environment. Several of them were detected from more than one model and environment, supporting their contribution to the trait. Notably, several of these common markers were detected in regions of chromosome 1 and 6, highlighting different association hotspots that contribute to a high portion of SI variance. Interestingly, one of these hotspots in chromosome 6 covers the region of the QTLs *BpSI.III* and *BpLD.I*, supporting their involvement in the genetic control of SI resistance in the field [12]. Specifically, the DArT marker 3542707 that localized on chromosome 6 at position 265,236,008 and explained the 23.6% of SI variation, is pinpointed between the flanked DArT markers 3545955 and 3542026 of the QTL *BpSI.III*. In this case, the QTL associated with lower SI explained a PVE of 11.5% [12]. The same DArT marker is also included in the QTL *BpLD.I*; here, the QTL associated with a lower larval development explained 16.5% of the variation. Although MTAs contributing to weevil SI resistance were identified on all pea chromosomes, none of the other MTAs overlap with previously detected bruchid-resistance QTL. Accordingly, our study uncovered additional genomic regions that participate in resistance, which would be useful for breeding.

Examination of the location of the associated markers allow identifying putative candidate genes that might contribute to resistance. Previous studies on the molecular basis of weevil resistance in *Vigna* spp. identified two tandemly duplicated polygalacturonase-inhibitor protein (PGIP) family genes, *VrPGIP1* and *VrPGIP2*, responsible for resistance that was successfully transferred to mung bean [60,61,62]. Here, we identified 23 genes in the near vicinity or containing the markers significantly associated with weevil SI resistance. Several of these genes contained more than one associated marker, supporting the contribution of these regions to the trait and the involvement of these genes in the resistance (Table 5). Two of these genes encoding a BolA-like family protein and an endoglucanase-12 like protein were mapped within the confidence interval region of *BrSI.III* (Table 5) [12], the former also co-localizing within *BrLD.I* confidence interval. In other species, *BolA-*like proteins were found to play a repressive role in the tolerance against excess iron and paraquat-induced oxidative stress in plants [32]. In addition, several of the candidate genes play an important role in pollen and seed development, including the genes 127136761, 127091639, 127120182, and 127118040 (Table 5). The first two genes, located 300 kb apart on chromosome 6 and tagged by three and two associated markers, respectively, encode a squalene epoxidase and an MYB-like transcription factor, which are important for embryo, seed, and seedling development [29,30,31]. In addition, gene 127120182, located on chromosome 1, is an ortholog of the *M. truncatula ZntB* proteins, implicated in Zn^2+^ uptake—a critical micronutrient for flowering and pollen formation [63]. The other candidate genes were shown to encode for homologs of genes that have been related to ion transport and stress response in other species, including genes 127083294 and 127119921 encoding an ABC transporter G family-like protein and a low-affinity inorganic phosphate transporter [64,65] and the genes 127117519, 127119769, 127120878, 127084927, and 127107464 encoding two heat-shock proteins, a ribonuclease, a dipeptidyl aminopeptidase, and a serine carboxypeptidase-like protein, respectively (Table 5) [23,66,67,68]. These results open great opportunity to improve our understanding of the genetic basis of weevil resistance in pea.

Altogether, we identified several novel resistance sources that should contribute to improve the resistance level to weevil in the field. More importantly, we identified a set of 73 molecular markers along with 23 candidate genes that should facilitate the implementation of marker-assisted breeding to quicken the introgression of resistance into an elite cultivar in the near future.

## 4. Materials and Methods

### 4.1. Plant Material, Experimental Design, and Assessments

Plant material consisted of a diverse core collection of 324 *Pisum* spp. accessions from worldwide origins, previously assembled to capture a broad range of phenotypic and genetic characteristics [69]. It includes accessions previously studied for their potential resistance to various pea pests, including the pea weevil *B. pisorum* (e.g., accessions IFPI3250, IFPI3280, IFPI3330, IFPI2354, IFPI2348, PI268480, JI227, and PI505059) [9].

The pea core collection was tested under field conditions at Córdoba, Spain, in four environments, consisting of two sites during two growing seasons (2018–2019 and 2019–2020), known for significant weevil prevalence (Table 5). This site represents the hot/dry summer Mediterranean environment, a common form of the Mediterranean climate characterized by hot, dry summers and mild, wet winters, according to the Köppen–Geiger classification system [70]. Each growing environment was defined by the specific year and location. We utilized a 19 × 19 alpha lattice design for experiments, including check controls with three replications at each site. Each experimental unit comprised a single row of 1 m in length containing 10 seeds, with rows spaced 0.7 m apart. Sowing occurred from November to December each year, aligned with local agricultural practices, though sowing dates varied slightly between locations and seasons. Weed control was managed using pre-emergence application of aclonifen (60%) in all seasons, supplemented by post-emergence treatments of bentazon (48%), imazamox (2.24%), and cycloxydim (10%) in 2019. In 2020, weed control was achieved solely through manual weeding.

Days from sowing till 50% flowering and 50% podding was estimated by continuous observations on the plots and referred to accumulated temperature terms, following the growing degree days (*GDD*) formula:

(1) $$GDD=\frac{T_{max}-T_{min}}{2}-T_{base}$$

$T_{max}$ is the daily maximum temperature, $T_{min}$ is the daily minimum temperature, and $T_{base}$ is the base temperature, which can vary depending on the crop (commonly 5 °C for pea). For each genotype, GDD_F is the accumulated *GDD* until days to flowering and GDD_P the accumulated *GDD* until days to podding, allowing categorization into early (1st quartile), intermediate (2nd and 3rd quartile), and late (4th quartile) maturity groups.

Upon reaching maturity, plants were harvested manually and threshed. This happened from late May to early June each season, depending on environmental conditions. To prevent bias in seed infestation evaluations, all accessions were processed, treated, and stored at 4 °C post harvest. SI was assessed by inspecting 100 randomly selected seeds per accession, with replication to detect weevil presence at the adult stage. Therefore, SI (%) was calculated as the ratio of infested seeds to the total number of evaluated seeds. To facilitate graphic comparisons among environments, SI values were expressed as SIr, referring to the average values of the susceptible cv. Messire within environment, which were established as 100% at each one [9].

### 4.2. Statistical Analysis

#### 4.2.1. Variance Analysis

To analyze the variance components within each environment for seed infestation (SI), we employed a linear mixed model. This model treated the genotype and the incomplete block nested within the replicate as random effects, while each complete replicate within an environment was considered a fixed effect. Broad-sense heritability (H^2^) was estimated using the formula from Toker et al. (2004) [71], defined as the ratio of genotypic variance to phenotypic variance. This approach also facilitated the calculation of the predicted means for each genotype, termed best linear unbiased predictors (BLUPs), which were used in subsequent analyses.

Additionally, a second linear mixed model was applied using a multi-environment trial (MET) framework. In this model, both the genotype and genotype–environment interaction (GEI) were treated as random effects, with all other sources of variance modelled as fixed effects. This model enabled the estimation of BLUPs for SI across the four environments, representing the GEI-independent component. Heritability in the MET model was calculated as the genotypic variance divided by the sum of the genotypic variance, GEI variance, and residual variance.

Both models and their respective formulas were implemented using R version 4.2.2 [72] with the metan package [73].

#### 4.2.2. Genotype Plus Genotype–Environment Interaction (GGE) Model

To analyze the combined effects of genotype (G) and genotype–environment interaction (GEI), we utilized a GGE biplot analysis, following the methodology proposed by Yan and Holland (2010) [74]. This model creates a biplot based on the first two principal components (PC1 and PC2), which are obtained through symmetric scaling and singular-value decomposition (SVD) of MET data centered around environmental means. The resulting GGE biplot allows for the simultaneous visualization of genotype performance and environmental interaction, facilitating the identification of superior genotypes and the classification of mega-environments [75]. GGE biplots are particularly valuable for comparing multiple genotypes across varied environments, aiding in the visualization of genotype stability and adaptability [76,77]. In our analysis, the ideal genotype displays low seed infestation (SI) values and consistent performance across all environments, indicative of minimal GEI. The proximity of environment scores to the origin in the biplot indicates smaller genotype differentials due to environmental variation.

The GGE model was estimated using the metan package in R. According to Yan and Kang (2002) [78], the model accommodates multiple data centering, scaling, and singular-value partitioning methods. Our GGE model configuration opted for an environment-centered approach without data scaling to preserve the relative magnitude of G and GEI effects. The which-won-where biplot generated identifies “vertex genotypes”, which are highly responsive to specific environments, as indicated by their vector lengths. A genotype at the origin would exhibit uniform performance across environments, showing no responsiveness to any environmental conditions.

#### 4.2.3. Non-Metric Multi-Dimensional Scaling Ordination (NMDS)

To evaluate the impact of environmental factors on SI, we conducted an NMDS, as described by Kruskal (1964) [28]. This analysis included 27 climate variables, which are detailed in Table S1. These variables were sourced from the Junta de Andalucía’s agroclimatic information network (https://www.juntadeandalucia.es/agriculturaypesca/ifapa/riaweb/web, accessed on 25 March 2024) and encompassed average, minimum, and maximum temperatures; average, minimum, and maximum humidity levels; accumulated radiation; evapotranspiration; and accumulated rainfall for three distinct growth stages: pre-flowering, flowering, and post flowering.

We performed 100 iterations of the NMDS to ensure the stability of the results and avoid the risk of capturing a local minimum stress, thereby increasing the likelihood of identifying the global minimum. Each iteration began with a different random starting configuration. From these iterations, we selected the two-dimensional solution that exhibited the lowest stress level, indicating the best representation of data structure in reduced dimensions. This analysis was performed using the vegan package in R Version 2.6-4 [79].

#### 4.2.4. Genotyping and Genome-Wide Analysis

The process of DNA extraction, DArT-Seq sequencing, and marker assembly, cleaning, and mapping were detailed in Rispail et al. (2023) [69]. Briefly, the genotyping of the pea core collection was conducted using the DArT-Seq method described by Barilli et al. (2018) [80]. DNA was extracted from pooled leaves of 20 seedlings per accession and analyzed using the high-density pea DArT-Seq 1.0 array. Subsequent data cleaning involved removing markers of low quality or without polymorphism, adhering to protocols recommended by Pavan et al. (2020) [81]. This process yielded 26,045 polymorphic Silico-DArT markers, which were mapped onto the two available *Pisum* reference genome sequences [82,83].

A genome-wide association study (GWAS) utilized these markers across 324 accessions, employing the BLUPs calculated for each environment and over the four tested environments (i.e., GEI-independent part). We performed the GWAS using single-trait analyses in both a single-locus mixed linear model (MLM) and a multi-locus Bayesian information and linkage disequilibrium iteratively nested keyway (BLINK) model [84]. These analyses conducted using GAPIT 3.0 [85] aimed to capture both major and minor genetic effects influencing SI variation. Population structure was controlled using a Bayesian information criterion (BIC)-based model selection procedure to determine the optimal number of principal components (PCs), following recommendations by Osuna-Caballero et al. (2024) [27]. Relatedness among individuals was accounted for using the Astle kinship matrix [86]. Marker significance was determined using a Bonferroni-corrected LOD threshold (− log10(0.05/number of markers)) and a variable LOD based on the false-discovery rate (FDR) method to detect markers that might be overlooked by the stringent Bonferroni threshold. This variable FDR threshold was estimated with the Qvalue R package and adjusted to ensure < 1 false positive Version 2.30.0 [87,88]. Linked DArT-Seq markers were identified as those within 2.5 kb and having a linkage disequilibrium (r^2^) of ≥0.5. Genomic inflation or deflation was assessed through the lambda (λ) statistic using the QQperm R package Version 1.0.1 [89]. Models showing a lambda value outside the range of 0.8 to 1.2 were excluded. Manhattan and Q-Q plots were generated using the CMplot R package 4.5.1 [90] to facilitate the visualization of the GWAS results.

Candidate genes were identified based on their proximity to significant markers or linkage disequilibrium with them (r^2^ > 0.5) within a 20 kb radius. These genes were located using the pea ZW6 reference genome browser [82]. Nucleic sequences of associated markers were submitted to the BLASTx algorithm to find homolog proteins in related species such as peanut (*Arachis hypogea*), common bean (*Phaseolis vulgaris*), soybean (*Glycine max*), *Medicago truncatula*, and the model species *Arabidopsis thaliana*. The functions of these candidate genes were inferred from annotations, the literature, and in silico analysis.

## Figures and Tables

**Table 1:** Descriptive statistics of weevil SI for each environment.

ENV	av.dev.	CV	Kurtosis	Mean	Median	min	max	Range	SE	Skewness
Agrario19	4.52	122.56	12.72	5.60	4.00	0.00	56.00	56.00	0.22	2.96
Agrario20	7.08	114.99	14.10	8.98	6.00	0.00	88.00	88.00	0.33	2.91
Puente19	7.36	100.59	2.30	9.18	7.00	0.00	68.00	68.00	0.30	1.32
Puente20	8.42	104.38	5.16	11.16	8.86	0.00	70.00	70.00	0.37	1.94

**Table 2:** Variance components obtained through the mixed model in the four environments, testing the percentage of weevil SI.

Parameters	Agrario19	Agrario20	Puente19	Puente20	BLUP_MET
Gen%	57.8	73.5	74.7	60.6	34.2
rep:block%	1.66	1.44	0	1.73	1.34
Res%	40.5	25.1	25.3	37.7	31.0
Phen_var	0.02	0.03	0.03	0.04	0.03
H^2^	0.58	0.74	0.75	0.61	0.34
H^2^mg	0.81	0.89	0.90	0.82	0.76
GEIr^2^	-	-	-	-	0.33
r_ge_	-	-	-	-	0.51
Accuracy	0.90	0.95	0.95	0.91	0.87
CV_g_	53	58	57.3	51.6	39.6
CV_r_	44.4	33.9	33.3	40.7	38.2
CV_ratio_	1.19	1.71	1.72	1.27	1.04

**Table 3:** Characteristics of the most resistant accessions within IAS pea core collection. Estimated SIr means (i.e., BLUP values) are presented in each location column. SIr = relative to the susceptible cv. Messire, established at 100%.

#	Bank Code	Taxa	Origen	Material	Flower Color	Seed Color	SIr (%) Agrario19	SIr (%) Agrario20	SIr (%) Puente19	SIr (%) Puente20
311	IFPI3261	*P. fulvum*	Syria	Wild	Orange	Black	22	8	6	18
249	PI344006	*P. s.* ssp. *elatius*	Greece	Wild	Purple	Brown	16	11	6	19
103	JI262	*P. s.* ssp. *elatius*	Turkey	Wild	Purple	Brown	16	15	6	15
309	IFFPI3257	*P. fulvum*	Syria	Wild	Orange	Black	21	15	6	14
248	PI344005	*P. s.* ssp. *elatius*	Greece	Wild	Purple	Brown	21	12	6	17
110	JI196	*P. s.* ssp. *humile*	Georgia	Land.	Purple	Brown	20	10	6	18
97	PI595933	*P. fulvum*	Australia	Wild	Orange	Brown	16	14	6	18
305	IFPI3232	*P. fulvum*	Syria	Wild	Orange	Black	20	12	6	19
269	JI524	*P. s.* ssp. *elatius*	Ethiopia	Wild	Purple	Brown	18	15	6	17
246	PI273209	*P. s.* ssp. *elatius*	Russia	Land.	Purple	Orange brown	21	12	6	17
197	PI357289	*P. s.* ssp. *sativum*	N. Maced.	Cult.	White	Green	22	12	6	19
244	PI173055	*P. s.* ssp. *elatius*	Turkey	Land.	Lilac	Brown	22	10	6	22
250	PI343976	*P. s.* ssp. *elatius*	Turkey	Wild	Purple	Orange brown	18	16	6	22
190	PI273207	*P. s.* ssp. *elatius*	Bulgaria	Land.	Purple	Orange brown	19	13	6	23
308	IFPI3253	*P. fulvum*	Syria	Wild	Orange	Black	21	15	6	22

**Table 4:** Candidate genes from GWAS-significant DArT markers. Gene ID displays the gene number in NCBI database as well as its name description in the second column. Chromosome number and physical position in pb is presented in the DArT position column. PVE represents the percentage of phenotypic variance explained by each marker in the model. The model with each environment configuration is in the model column, and the LOD threshold is displayed in the last column.

Gene ID	Gene Description	DArT Position	DArT ID	PVE	Model Configuration	LOD
127120182	Uncharacterized LOC127120182	Chr1_110,246,659	4656306	7.1	BLINK_Puente20	10.2
		Chr1_110,246,659	4656306	7.1	MLM_Puente20	4.9
127084996	Glycine-rich RNA-binding protein RZ1A	Chr1_458,055,252	3542530	4.1	BLINK_Puente20	7.8
		Chr1_458,055,252	3542530	4.1	MLM_Puente20	6.1
127083848	Uncharacterized LOC127083848	Chr1_453,262,700	3567504	1.2	BLINK_Puente20	11.1
		Chr1_453,262,700	3567504	1.9	MLM_BLUP_MET	4.8
		Chr1_453,262,700	3567504	1.2	MLM_Puente20	4.9
		Chr1_453,262,700	3567504	7.2	BLINK_Puente19	8.8
		Chr1_453,262,700	3567504	7.2	MLM_Puente19	4.5
127120989	Uncharacterized LOC127120989	Chr2_2,267,172	5938000	0.4	MLM_Agrario19	5.6
		Chr2_2,267,292	5939473	1.1	MLM_Agrario19	5.6
127117519	Uncharacterized LOC127117519	Chr2_4,167,178	3546634	2.2	MLM_Puente19	6.2
		Chr2_4,167,181	5937650	2.6	MLM_Puente19	6.3
127118040	Centromere/kinetochore protein zw10	Chr2_50,182,638	3556456	0.11	BLINK_Puente19	5.3
		Chr2_50,184,923	3558874	0.17	BLINK_Puente19	5.3
		Chr2_50,185,115	3558119	0.08	BLINK_Puente19	5.3
127119769	Heat-shock factor protein HSF8	Chr2_408,028,665	3547098	12.8	MLM_Puente19	6.2
		Chr2_408,028,667	3542446	14.3	BLINK_Puente19	6.8
		Chr2_408,028,667	3542446	14.3	MLM_Puente19	4.5
		Chr2_408,041,733	3558655	5.1	MLM_Puente19	6.2
127119921	ABC transporter G family member 14	Chr2_426,964,234	26138253	2.5	MLM_Puente19	6.8
		Chr2_426,964,229	5930625	2.6	MLM_Puente19	6.5
127120878	Ribonuclease MRP protein subunit POP4	Chr2_489,920,805	5940469	5.8	BLINK_Puente20	8.8
		Chr2_489,920,805	5940468	8.6	MLM_Puente20	6.0
127073915	Serine/threonine/tyrosine-protein kinase HT1	Chr4_119,335,846	3547803	10.7	BLINK_BLUP_MET	5.4
		Chr4_119,335,846	3547803	8.0	MLM_Agrario20	6.1
127083294	Low-affinity inorganic phosphate transporter	Chr5_234,162,185	3559664	0.52	BLINK_BLUP_MET	6.3
		Chr5_234,162,185	3559664	0.52	MLM_BLUP_MET	5.9
127084927	Uncharacterized protein LOC127084927	Chr5_380,528,488	3539562	2.6	MLM_Puente19	6.8
		Chr5_380,540,916	26137620	6.2	BLINK_Puente19	5.2
127136761	Squalene epoxidase	Chr6_241,397,688	3548086	13.6	BLINK_Puente19	4.8
		Chr6_241,397,688	3548086	13.6	MLM_Puente19	5.3
		Chr6_241,397,688	3548086	12.3	BLINK_BLUP_MET	6.8
		Chr6_241,399,701	5888303	4.3	BLINK_Puente19	6.7
		Chr6_241,399,704	5955249	5.7	MLM_Puente20	5.8
		Chr6_241,399,704	5955249	2.7	MLM_Puente19	7.1
127092211	BolA4, chloroplast/mitochondria	Chr6_265,236,008	3542707	23.6	MLM_BLUP_MET	5.7
		Chr6_265,236,008	5911996	18.1	BLINK_Puente19	6.8
127097033	60S ribosomal protein L14	Chr6_300,875,627	3550101	17.5	BLINK_Agrario19	5.6
		Chr6_300,875,627	3550101	19.4	BLINK_Puente20	6.1
		Chr6_300,875,630	3550101	30.8	BLINK_BLUP_MET	5.7
127091639	Transcription factor MYB SRM1	Chr6_241,700,843	5906703	6.1	BLINK _Agrario20	6.7
		Chr6_241,700,843	5906703	8.5	BLINK _BLUP_MET	5.7
		Chr6_241,700,843	5906703	5.4	MLM_Puente19	7.1
		Chr6_241,700,843	5906703	7.1	MLM_Puente20	6.1
		Chr6_241,700,840	5906695	6.0	MLM_Agrario20	6.7
		Chr6_241,700,840	5906695	8.0	MLM_BLUP_MET	5.5
		Chr6_241,700,840	5906695	4.8	BLINK _Puente19	6.8
		Chr6_241,700,840	5906695	7.1	MLM_Puente20	6.1
127097188	Endoglucanase 12-like	Chr6_259,693,325	41129807	1.0	MLM_Puente19	6.4
		Chr6_259,693,328	19759901	0.7	MLM_Puente19	6.4
127096593	Methyltransferase METTL6	Chr6_311,453,191	3552572	29.2	BLINK_BLUP_MET	9.0
		Chr6_311,453,191	3552572	22.9	MLM_Puente19	6.3
		Chr6_311,453,191	3552572	15.8	MLM_Puente20	6.1
127097503	Heavy metal-associated isoprenylated plant protein	Chr6_427,158,133	4662095	4.7	BLINK_Agrario19	5.6
		Chr6_427,158,135	4660398	4.8	BLINK_Agrario19	5.6
127105338	Protein MAIN-LIKE 1	Chr7_288,124,543	3566185	5.3	BLINK_Agrario20	5.8
		Chr7_288,124,543	3566185	8.1	BLINK_BLUP_MET	5.5
127107464	Serine carboxypeptidase	Chr7_320,302,456	3549353	8.5	BLINK_BLUP_MET	6.9
		Chr7_320,302,459	3568172	4.4	BLINK_BLUP_MET	5.8
127105952	Uncharacterized protein LOC127105952	Chr7_506,057,986	4657153	17.3	BLINK_Agrario19	5.3
		Chr7_506,057,986	4657153	17.3	MLM_Agrario19	5.6
127101072	Non-specific lipid-transfer protein 3	Chr7_512,206,490	5923023	1.1	MLM_Puente20	6.1
		Chr7_512,206,488	5880896	2.0	MLM_Puente20	6.1

**Table 5:** Description of the environments tested in the multi-environment trials during the pea cycle from December to May.

ENV.	Season	Site (Decimal Degrees Coordinates)	Soil Type	Soil pH	Organic Matter (g/100 g)	Available Phosphorus (mg/kg)	Average T_max_ (°C)	Average T_min_ (°C)	Rain (mm)
Agrario19	2018–2019	37.856833, −4.802690	Cambisol	7.5	1.2	15.1	22.2	6.3	206
Agrario20	2019–2020	37.860775, −4.799413	Cambisol	-	-	-	20.1	6.6	363
Puente19	2018–2019	37.864470, −4.789733	Vertisol	7.8	0.7	9.9	21.1	5.8	127
Puente20	2019–2020	37.866372, −4.787661	Vertisol	-	-	-	21.0	8.6	382

## Data Availability

The DArTseq marker datasets analyzed during the current study are available in the Zenodo repository, https://zenodo.org/records/7180467 (accessed on 10 October 2023). The phenotypic datasets generated and analyzed are available in the GitHub repository, https://github.com/SalvaOsuna/Weevil-pea.git (accessed on 15 January 2024).

## Supplementary Materials

The following supporting information can be downloaded at: https://www.mdpi.com/article/10.3390/ijms25147920/s1.
