# Supplementary material for: Genome-Wide Association Studies on Resistance to Pea Weevil: Identification of Novel Sources of Resistance and Associated Markers
Source: Int J Mol Sci. 2024 Jul 19;25(14):7920. doi: 10.3390/ijms25147920 (PMC11276686; doi:10.3390/ijms25147920)
Supplement: Supplementary file 1 [file ijms-25-07920-s001.zip › Table S1.pdf]

Table S1. Climate variables including: maximum temperature (Tmax), minimum temperature (Tmin), average temperature (TAve), maximum humidity (Hmax), minimum humidity (Hmin), average humidity (HAve), Radiation (Rad), rain and Evapotranspiration (ETo) during different growing stages pre-flowering (Pre), flowering (Flow) and post-flowering (Post) characterizing the 4 environments (combination of location and season) of the trials.

ENV	Pre_Tmax	Pre_Tmin	Pre_Tave	Pre_Hmax	Pre_Hmin	Pre_Have	Pre_Rad	Pre_Rain	Pre_Eto	Flow_Tmax	Flow_Tmin	Flow_Tave	Flow_Hmax	Flow_Hmin	Flow_Have	Flow_Rad	Flow_Rain	Flow_Eto	Post_Tmax	Post_Tmin	Post_Tave	Post_Hmax	Post_Hmin	Post_Have	Post_Rad	Post_Rain	Post_Eto
**Agrario19**	17.24	3.66	9.41	99.57	47.44	82.23	1229	148.8	143.2	23.49	5.47	13.97	96.35	28.51	66.66	225.6	0.4	33.47	25.96	9.68	17.86	90.27	28.56	58.21	1819.3	57.2	360.5
**Agrario20**	16.89	6.17	10.74	98.71	57.38	86.7	839.6	271.8	120.8	22.17	5.12	12.44	99.4	31.98	75.62	177.8	0.2	27.44	21.11	8.66	14.35	98.92	43.17	77.32	810.6	91.2	146
**Puente19**	17.48	3.12	9.22	99.54	45.17	81.1	1212	71.4	140.2	22.35	4.55	13.28	94.65	24.09	59.46	237.5	0.2	36.34	26.28	9.97	18.17	90.46	29.04	58.57	1701.2	57	340.5
**Puente20**	16.79	6.18	10.7	98.69	57.61	86.76	809.8	271.8	116.9	22.04	5.47	12.48	99.41	34.65	77.99	173.1	0.2	25.95	21.16	8.46	14.28	98.94	42.73	77.07	571.6	50.2	82.82
