# Supplementary material for: Genome-Wide Association Studies on Resistance to Pea Weevil: Identification of Novel Sources of Resistance and Associated Markers
Source: Int J Mol Sci. 2024 Jul 19;25(14):7920. doi: 10.3390/ijms25147920 (PMC11276686; doi:10.3390/ijms25147920)
Supplement: Supplementary file 1 [file ijms-25-07920-s001.zip › Table S3.pdf]

Table S3. Full list of associated DArT markers. Chr refers to the Chromosome; Pos to the physical position in pb; P.value the significance of the association; MAF is the minor allele frequency; nobs is the number of observations; HBP value refers to Benjamini-Hochberg Procedure; Effect size; Model and Trait configuration; expFP are the significance values from the False Discovery Rate; LOD threshold; and criteria of the selection marker.

DArT_ID	Chr	Pos_Cameor	Pos_ZW6	P.value	MAF	nobs	H.B.P.Value	Effect	Model	Trait	expFP	LOD	Criterion
4656306	1	16797281	110246659	1.97E-10	0.13	320	1.71E-06	-0.04875217	BLINK	Puente20	5.14E-06	10.2	Gapit Default
4656306	1	16797281	110246659	1.18E-05	0.13	320	3.07E-01	-0.05230086	MLM	Puente20	3.00E-01	4.9	FDR adjusted
8173549	1	191324161	274543094	3.35E-05	0.47	320	1.96E-02	0.03050874	MLM	Puente19	7.49E-01	6.2	Gapit Default
8174271	1	191344682	274563975	2.16E-05	0.49	320	1.73E-02	0.03089771	MLM	Puente19	4.78E-01	6.2	Gapit Defaul
3542530	1	367307446	458055252	1.43E-07	0.33	320	4.50E-04	0.02861752	BLINK	Puente20	3.60E-03	7.8	Gapit Default
3542530	1	367307446	458055252	6.60E-06	0.33	320	2.86E-02	0.04075247	MLM	Puente20	1.34E-01	6.1	Gapit Default
3567504	1	NA	453262700	1.56E-11	0.12	320	2.03E-07	-0.05334152	BLINK	Puente20	4.06E-07	11.1	Gapit Default
3567504	1	NA	453262700	5.32E-05	0.12	320	4.60E-01	-0.03770617	MLM	BLUP_MET	8.99E-01	4.8	FDR adjusted
3567504	1	NA	453262700	5.05E-05	0.12	320	3.67E-01	-0.0662529	MLM	Puente20	7.18E-01	4.9	FDR adjusted
3567504	1	NA	453262700	3.05E-09	0.12	320	3.97E-05	-4.91E-02	BLINK	Agrario19	7.60E-05	8.8	Gapit Default
3567504	1	NA	453262700	7.72E-05	0.12	320	7.73E-01	-4.98E-02	MLM	Agrario19	7.73E-01	4.5	FDR adjusted
5938000	2	1840404	2267172	4.09E-05	0.18	320	7.61E-02	-0.02923866	MLM	Agrario19	8.65E-01	5.6	FDR adjusted
5939473	2	1840524	2267292	4.00E-05	0.15	320	7.61E-02	-0.03015059	MLM	Agrario19	8.04E-01	5.6	FDR adjusted
3546634	2	NA	4167178	2.44E-05	0.16	320	1.73E-02	0.04155048	MLM	Puente19	5.56E-01	6.2	Gapit Default
5937650	2	NA	4167181	1.89E-05	0.19	320	1.64E-02	0.03898567	MLM	Puente19	4.38E-01	6.3	Gapit Default
3556456	2	32064080	50182638	1.24E-05	0.37	320	1.27E-01	0.03021919	BLINK	Puente19	2.55E-01	5.3	FDR adjusted
3558874	2	32066364	50184923	1.96E-05	0.37	320	1.27E-01	0.02906825	BLINK	Puente19	5.09E-01	5.3	FDR adjusted
3558119	2	32066584	50185115	1.47E-05	0.38	320	1.27E-01	0.02902622	BLINK	Puente19	3.82E-01	5.3	FDR adjusted
3547098	2	357742505	408028665	2.42E-05	0.19	320	1.73E-02	-0.04170348	MLM	Puente19	5.25E-01	6.2	Gapit Default
3542446	2	357742507	408028667	1.06E-06	0.16	320	4.24E-03	-0.04918406	MLM	Puente19	1.89E-02	6.8	Gapit Default
3542446	2	357742507	408028667	1.14E-04	0.16	320	8.19E-01	-0.04752538	MLM	Puente19	8.14E-01	4.5	FDR adjusted
3558655	2	357755566	408041733	2.47E-05	0.25	320	1.73E-02	-0.03465181	MLM	Puente19	5.71E-01	6.2	Gapit Default
26138253	2	374513680	426964234	1.63E-06	0.08	320	4.24E-03	0.06249317	MLM	Puente19	3.77E-02	6.8	Gapit Default
5930625	2	374513683	426964229	5.32E-06	0.09	320	9.89E-03	0.05789059	MLM	Puente19	1.23E-01	6.5	Gapit Default
5940469	2	scaffold00769	489920805	8.95E-09	0.09	320	4.18E-05	0.05869077	BLINK	Puente20	2.09E-04	8.8	Gapit Default
5940468	2	scaffold00769	489920805	1.93E-05	0.06	320	3.60E-02	-0.06030726	MLM	Puente20	3.93E-01	6.0	Gapit Default
3547803	4	scaffold00043	119335846	2.23E-05	0.27	320	1.16E-01	-0.01265568	BLINK	BLUP_MET	5.63E-01	5.4	FDR adjusted
3547803	4	scaffold00043	119335846	5.60E-06	0.27	320	2.57E-02	-0.03559269	MLM	Agrario20	1.08E-01	6.1	Gapit Default

3559664	5	186908605	234162185	1.68E-06	0.45	320	1.46E-02	-0.01625212	BLINK	BLUP_MET	4.22E-02	6.3	Gapit Default
3559664	5	186908605	234162185	4.57E-06	0.45	320	3.77E-02	-0.02356615	MLM	BLUP_MET	6.35E-02	5.9	Gapit Default
3539562	5	330215504	380528488	5.24E-07	0.06	320	4.24E-03	0.07420482	MLM	Puente19	1.13E-02	6.8	Gapit Default
26137620	5	330228061	380540916	4.43E-05	0.33	320	1.81E-01	-0.02991921	BLINK	Puente19	9.04E-01	5.2	FDR adjusted
3562194	5	459536097	253443965	1.43E-06	0.47	320	1.24E-02	-0.03898563	MLM	Agrario20	3.13E-02	6.4	Gapit Default
3562194	5	459536097	253443965	1.06E-04	0.47	320	9.92E-01	-0.03609441	MLM	Agrario20	9.85E-01	4.4	FDR adjusted
5919697	5	561660724	NA	9.95E-06	0.06	320	2.90E-02	0.06326173	MLM	Puente20	1.81E-01	6.1	Gapit Default
5886122	5	561660727		3.85E-05	0.07	320	5.57E-02	0.05427929	MLM	Puente20	7.81E-01	5.8	FDR adjusted
3548086	6	147983050	241397688	3.77E-05	0.41	320	4.43E-01	-0.02825938	BLINK	Puente19	8.85E-01	4.8	FDR adjusted
3548086	6	147983050	241397688	1.44E-05	0.41	320	1.45E-01	-0.04131701	FarmCPU	Puente19	2.91E-01	5.3	FDR adjusted
3548086	6	147983050	241397688	8.68E-06	0.41	320	5.80E-03	-0.02133111	MLM	BLUP_MET	1.66E-01	6.8	Gapit Default
3548086	6	147983050	241397688	1.23E-06	0.41	320	1.26E-02	-0.04131701	MLM	Puente19	2.29E-02	6.4	Gapit Default
3548086	6	147983050	241397688	1.00E-04	0.41	320	9.34E-01	-0.03795887	MLM	Puente19	9.34E-01	4.4	FDR adjusted
5888303	6	147985124	241399701	2.24E-06	0.07	320	5.22E-03	0.06810313	MLM	Puente19	5.11E-02	6.7	Gapit Default
5955249	6	147985127	241399704	1.67E-05	0.06	320	5.45E-02	0.06389102	MLM	Agrario20	3.67E-01	5.8	FDR adjusted
5955249	6	147985127	241399704	1.60E-07	0.06	320	2.09E-03	0.07850611	MLM	Puente19	3.72E-03	7.1	Gapit Default
3542707	6	168525220	265236008	2.51E-05	0.43	320	6.55E-02	-0.02223637	MLM	BLUP_MET	5.52E-01	5.7	FDR adjusted
5911996	6	168525220	265236008	1.36E-06	0.07	320	4.24E-03	0.07049302	MLM	Puente19	3.02E-02	6.8	Gapit Default
3550101	6	208566056	300875627	3.43E-05	0.49	320	7.61E-02	-0.02516201	MLM	Agrario19	6.80E-01	5.6	FDR adjusted
3550101	6	208566056	300875627	4.76E-06	0.49	320	2.48E-02	-0.03809869	MLM	Puente20	9.66E-02	6.1	Gapit Default
44299313	6	208566059	300875630	2.38E-05	0.44	320	6.55E-02	0.01818125	MLM	BLUP_MET	4.97E-01	5.7	FDR adjusted
5906703	6	251955535	241700843	4.42E-07	0.08	320	5.79E-03	0.06370397	MLM	Agrario20	4.87E-03	6.7	Gapit Default
5906703	6	251955535	241700843	2.16E-05	0.08	320	6.55E-02	0.03763309	MLM	BLUP_MET	4.41E-01	5.7	FDR adjusted
5906703	6	251955535	241700843	1.05E-07	0.08	320	2.09E-03	0.06891317	MLM	Puente19	1.86E-03	7.1	Gapit Default
5906703	6	251955535	241700843	9.15E-06	0.08	320	2.90E-02	0.06236882	MLM	Puente20	1.58E-01	6.1	Gapit Default
5906695	6	251955538	241700840	4.45E-07	0.08	320	5.79E-03	0.0628987	MLM	Agrario20	9.75E-03	6.7	Gapit Default
5906695	6	251955538	241700840	4.23E-05	0.08	320	8.86E-02	0.03654508	MLM	BLUP_MET	8.21E-01	5.5	FDR adjusted
5906695	6	251955538	241700840	7.23E-07	0.08	320	4.24E-03	0.0634124	MLM	Puente19	1.51E-02	6.8	Gapit Default
5906695	6	251955538	241700840	4.26E-06	0.08	320	2.48E-02	0.06470248	MLM	Puente20	7.73E-02	6.1	Gapit Default
41129807	6	262348100	259693325	6.44E-06	0.24	320	1.05E-02	0.03989952	MLM	Puente19	1.49E-01	6.4	Gapit Default
19759901	6	262348103	259693328	9.80E-06	0.22	320	1.28E-02	0.03989349	MLM	Puente19	2.17E-01	6.4	Gapit Default
3552572	6	283035925	311453191	9.55E-10	0.47	320	2.49E-05	-0.02168528	BLINK	BLUP_MET	2.41E-05	9.0	Gapit Default

3552572	6	283035925	311453191	1.49E-05	0.47	320	1.49E-02	-0.0379095	MLM	Puente19	3.45E-01	6.3	Gapit Default
3552572	6	283035925	311453191	1.10E-05	0.47	320	2.90E-02	-0.03724168	MLM	Puente20	2.03E-01	6.1	Gapit Default
4662095	6	392096645	427158133	3.09E-05	0.29	320	7.61E-02	-0.02313161	MLM	Agrario19	6.18E-01	5.6	FDR adjusted
4660398	6	392096647	427158135	2.16E-05	0.29	320	7.61E-02	-0.02350086	MLM	Agrario19	3.71E-01	5.6	FDR adjusted
3566185	7	244194637	288124543	1.18E-05	0.49	320	4.39E-02	-0.03192395	MLM	Agrario20	2.58E-01	5.8	Gapit Default
3566185	7	244194637	288124543	4.42E-05	0.49	320	8.86E-02	-0.01854723	MLM	BLUP_MET	9.70E-01	5.5	FDR adjusted
3549353	7	362192113	320302456	1.59E-07	0.29	320	4.15E-03	-0.02756249	MLM	BLUP_MET	3.50E-03	6.9	Gapit Default
3568172	7	362192124	320302459	1.29E-05	0.43	320	5.11E-02	-0.01893336	MLM	BLUP_MET	2.59E-01	5.8	FDR adjusted
4657153	7	456108375	506057986	2.79E-05	0.40	320	1.46E-01	-0.01949259	BLINK	Agrario19	7.28E-01	5.3	FDR adjusted
4657153	7	456108375	506057986	4.98E-05	0.40	320	7.77E-02	-0.02643204	MLM	Agrario19	9.47E-01	5.6	FDR adjusted
5923023	7	464771058	512206490	3.05E-06	0.07	320	2.48E-02	0.06876431	MLM	Puente20	5.79E-02	6.1	Gapit Default
5880896	7	464771060	512206488	2.12E-06	0.07	320	2.48E-02	0.07029013	MLM	Puente20	1.93E-02	6.1	Gapit Default
